# Synthesis and Characterization of Corn Stover-Based Cellulose Triacetate Catalyzed by Ionic Liquid Phosphotungstate

**DOI:** 10.3390/ijms23126783

**Published:** 2022-06-17

**Authors:** Xiwen Jia, Dongyi Guo, Qingjiang Yan, Haitao Yu, Qian Lyu, Lujia Han, Chengfeng Zhou, Weihua Xiao

**Affiliations:** 1 College of Engineering, China Agricultural University, Beijing 100083, China; xwjia2016@126.com (X.J.); s20193071183@cau.edu.cn (D.G.); yanqingjiang@aliyun.com (Q.Y.); yht@cau.edu.cn (H.Y.); lvqian19940518@163.com (Q.L.); hanlj@cau.edu.cn (L.H.); 2 State Key Laboratory of Bio-Fibers and Eco-Textiles, Qingdao University, Qingdao 266071, China; chengfengzhou@qdu.edu.cn

**Keywords:** corn stover, ionic liquid phosphotungstate, cellulose triacetate, synthesis, physicochemical properties

## Abstract

Cellulose triacetate (CTA) was successfully synthesized from corn stover cellulose (CSC) in the presence of [PyPS]_3_PW_12_O_40_ (IL-POM). The effects of IL-POM contents, reaction temperature, and reaction time on the yield and degree of substitution of CTA were investigated. The synthesized CTA was characterized by SEM, FTIR, and TGA, and the degree of polymerization and solubility in various organic solvents were evaluated. Results showed that the optimum reaction conditions were as follows: 0.04 g of IL-POM, reaction temperature of 140 °C, and reaction time of 45 min, for 0.4 g of CSC and 9 mL of glacial acetic acid. The yield of CTA under optimum reaction conditions was as high as 79.27%, and the degree of substitution was 2.95. SEM and FTIR results showed that the cellulose acetylation occurred, and CTA was synthesized. The TGA results revealed that the decomposition temperature of CTA increased by about 30 °C when compared with that of CSC. A simple, environment-friendly, and efficient process for the preparation of CTA from CSC was constructed, which provides a new pathway for the high-value utilization of corn stover.

## 1. Introduction

In recent years, the efficient utilization of agricultural residues has become the focus of discussion. Agricultural residues are considered as abundant, cheap, and easily available natural biomass resources [1,2]. As the residue of important grain crops, corn stover is an important biomass resource with the advantages of abundant reserves, low cost, and easy access. Corn stover can be transformed into high-value chemicals, which can realize the efficient utilization of resources, and is of great significance to alleviating the fossil energy crisis and solving environmental pollution [3,4].

Cellulose is the most abundant natural polysaccharide and the main constituent (30–50%) of corn stover. Cellulose consists of *β*-(1→4)-linked d-glucose units, which result in cellulose’s high crystallinity (over 40%) and difficulty in dissolving in water and other common solvents [5,6,7,8]. Interestingly, cellulose can be modified into cellulose derivatives through partial esterification or etherification of the hydroxyl groups on the backbone [9]. These cellulose derivatives, including methyl cellulose [10], carboxymethyl cellulose [11], and cellulose acetate [12], are easier to dissolve and process compared to the native cellulose.

Cellulose acetate is one of the important organic acid esters in cellulose; cellulose triacetate (CTA) refers to cellulose acetate with a degree of substitution (DS) between 2.7 and 3.0 [12]. CTA is often used in reverse osmosis membranes, aerospace materials, and textile fibers because of its advantages of good thermoplasticity, strong mechanical properties, and excellent optical properties [13,14,15]. Most traditional CTA preparation methods use natural wood pulp or cotton pulp as the main raw material, which is synthesized by sulfuric acid catalysis. Although CTA synthesis catalyzed by sulfuric acid is widely used, the use of sulfuric acid has the drawbacks of being ecologically unfriendly and causing considerable corrosion to equipment [16]. Furthermore, residual sulfate can reduce the thermal stability of CTA and affect its application. Recently, Nemr et al. [17] reported the preparation of cellulose triacetate using iodine as a catalyst; however, iodine as a catalyst could not be recycled, and a reducing agent needed to be added to remove it at the end of the reaction, which increased the preparation cost. Moreover, the yield was relatively low. Therefore, it is necessary to identify or develop an environment-friendly acid catalyst with high catalytic efficiency and recyclability.

During the past decades, the application of heteropolyanion-based ionic liquid (IL-POM) catalysts (including phosphotungstic acid, silicotungstic acid, and phosphomolybdic acid) in the field of catalytic reactions such as esterification, hydrogenation, and oxidation has received extensive attention due to their excellent catalytic performance and the consideration of environmental protection [18,19,20]. It is noteworthy that phosphotungstic acid has stronger Brønsted acidity than sulfuric acid; thus, it can show stronger catalytic activity than some traditional inorganic acids in some acid catalytic systems [21]. Meanwhile, owing to their inherent structures and physiochemical properties, IL-POMs are usually not miscible with polar solvents at room temperature but miscible at high temperatures [22]. Therefore, IL-POMs can be used as homogeneous catalysts at appropriate temperatures and then precipitate into a gelatinous solid at low temperatures, providing significant convenience for the separation and recovery of catalysts [19]. The use of IL-POMs is an effective and sustainable method for the conversion of cellulose and lignocellulosic biomass [23], and most of the current research on the esterification reaction focuses on the catalytic conversion of cellulose to levulinic acid or levulinic acid ester using IL-POM [19,22,24]. However, there are few studies on the catalytic synthesis of CTA from corn stover cellulose using an IL-POM catalyst.

The objective of the present study was to convert corn stover cellulose (CSC) into CTA using [PyPS]_3_PW_12_O_40_ (IL-POM). The effects of the content of IL-POM, reaction time, and temperature on the yield and degree of substitution of CTA were investigated. The structural characteristics, micromorphology, and thermal stability of CTA obtained under the optimal conditions were characterized by FTIR, SEM, and TGA, respectively. Furthermore, the degree of polymerization of CTA and its solubility in common organic solvents were analyzed and discussed. This study is expected to provide a reference for the efficient synthesis and high-value utilization of corn stover cellulose acetate.

## 2. Materials and Methods

### 2.1. Materials and Chemicals

Corn stover was collected from Huaian County, Hebei Province, China. After collection, the corn stover was air-dried and milled through a 40-mesh screen using an RT-34 milling machine (Rong Tsong Precision Technology, Hong Kong, China). The milled sample was stored in a desiccator at room temperature (25 °C). The chemical composition of corn stover was 36.94% cellulose, 21.10% hemicellulose, 18.28% lignin, and 2.43% ash. Peracetic acid was purchased from Shanghai HABO Chemical Technology Co., Ltd. (Shanghai, China). Maleic acid, pyridine, and acetic anhydride were obtained from Beijing Chemical Plant Co., Ltd. (Beijing, China). 1,3-Propanesulfonate and acetic acid were supplied by Shanghai Macklin Biochemical Co., Ltd. (Shanghai, China). Phosphotungstic acid (H_3_PW_12_O_40_) was purchased from China National Pharmaceutical Group Corporation (Beijing, China). All other reagents and chemicals were of analytical grade.

### 2.2. Preparation of Cellulose from Corn Stover

Referring to the previous research [25], PAM (peracetic acid and maleic acid) pretreatment was conducted in a Milestone apparatus equipped with an internal temperature sensor and a magnetic stirrer. First, 4 g of corn stover sample was loaded into a sealed Teflon reaction vessel, followed by the addition of 160 mL of PAM solution. The mixture was heated to 130 °C within 3 min for the designated residence time. The reaction time was 60 min. The concentrations of peracetic acid (1.5 wt.%) and maleic acid (3.0 wt.%) in the PAM solution were determined, as well as a solid–liquid (s/l) ratio of 1:20 (*w*/*v*). After pretreatment, the vessel was cooled to room temperature (25 °C) by immersion in water. Separation of suspension was conducted in a Buchner funnel. The wet solid residue was washed with deionized water until the pH was nearly neutral. The residue was dried in an oven at 105 °C for use, and it was denoted as corn stover cellulose (CSC). The dried product was crushed and passed through a 40-mesh screen for use.

### 2.3. Synthesis of IL-POM Catalyst

The general preparation process of the IL-POM catalyst was as follows [26]: firstly, pyridine (0.11 mol) and 1,3-propanesulfonate (0.10 mol) were dissolved in toluene and stirred vigorously at 50 °C for 24 h under the protection of nitrogen; then the white precipitated 1,3-propanesultone-pyridine (PyPS) mixture was collected by filtration, washed with ether (100 mL each time, three times), and eventually dried in vacuo overnight. PyPS (0.06 mol, 12.08 g) was added to an aqueous solution containing phosphotungstic acid (H_3_PW_12_O_40_) (0.02 mol). The mixture was vigorously stirred at room temperature for 24 h and then vacuum-dried to obtain the final product [PyPS]_3_PW_12_O_40_ (IL-POM). The dried product was ground and passed through a 60-mesh screen for use.

### 2.4. Synthesis of Cellulose Triacetate (CTA)

**Scheme 1:**
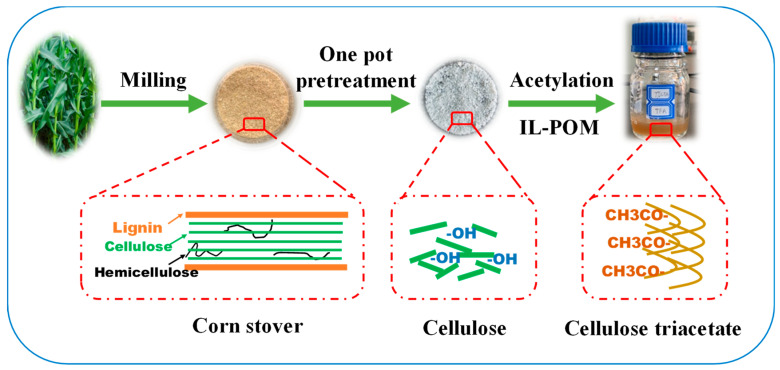
Schematic presentation of the process of preparing cellulose triacetate (CTA) from corn stover.

**Scheme 2:**
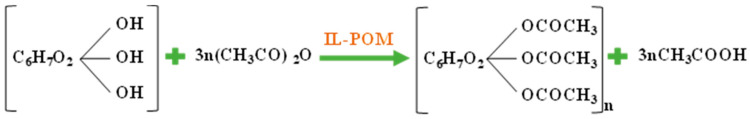
Reaction equation for synthesizing CTA from cellulose catalyzed by IL-POM.

The CSC (0.4 g) was placed into a special quartz tube for the microwave digestion instrument, and a certain amount of IL-POM catalyst was added to the quartz tube. Then, acetic acid (9 mL) and acetic anhydride (2 mL) were added. The quartz tube was placed on the special reaction rack and covered with a Teflon lid. The above reaction system was placed into a special Teflon tank filled with 350 mL of deionized water and then into a microwave digestion apparatus. High-purity nitrogen (2 MPa) was loaded, and the reaction was carried out at a certain reaction temperature for a certain period of time. The product was transferred to a centrifuge tube and centrifuged at 8000 r/min for 30 min. Then, the centrifuged liquid was poured into deionized water for precipitation, and then filtered and washed. The washed solids were placed into a beaker filled with deionized water, and ultrasonicated for 4 h. The solid in the beaker was filtered and washed, followed by drying at 105 °C for 12 h in an oven to obtain CTA. Schematic diagrams of the CTA synthesis and acetylation process from corn stover are shown in Scheme 1 and Scheme 2, respectively.

### 2.5. Determination of CTA Substitution Degree

The degree of substitution of cellulose acetate was determined according to ASTM D871-96 [27]. The specific methods were as follows: firstly, CTA (1.00 g) was weighed and placed it in a flat-bottom flask, which was rotated to evenly distribute the sample at the bottom of the beaker. Then, 60 mL of acetone was added, and the mixture was stirred for 30 min at a constant speed. Next, 15 mL of dimethyl sulfoxide was carefully added dropwise into the flask; the flask wall was rinsed, and the flask was stirred at a slow and uniform speed for 60 min to completely dissolve the sample. Next, 15 mL of NaOH (1.0 M) sodium hydroxide solution was added to the flask, which was stirred at a constant speed for 120 min. Then, 5–6 drops of phenolphthalein reagent were added, and the excess NaOH solution was titrated with 1.0 M H_2_SO_4_ standard solution. The solution was titrated at a constant stirring rate until the solution changed from pink to colorless and stopped within 1 min. Then, four more drops of 1.0 M H_2_SO_4_ standard solution were dropped and mixed evenly. The excess sulfuric acid was titrated with 0.1 M NaOH sodium hydroxide standard solution until the solution turned pale pink and remained unchanged. At the same time, a blank test was conducted. The acetyl content (AC%) and the degree of substitution (DS) of CTA were calculated using Equations (1) and (2), respectively.

(1) $$\mathrm{AC}\left(\%\right)=\left[\left(D-C\right)\mathrm{Na}+\left(A-B\right)\right]\mathrm{Nb}\times \frac{4.3005}{W},$$

where A is the sodium hydroxide solution to titrate the volume of the sample (mL), B is the volume of the sodium hydroxide solution titration blank (mL), Na is the molar concentration of the sodium hydroxide standard titration solution (mol/L), Nb is the molar concentration of the sulfuric acid standard titration solution (mol/L), C is the sulfuric acid calibration sample volume (mL), D is the sulfuric acid titration blank volume (mL), and W is the quality of the cellulose acetate sample (mL).

(2) $$\mathrm{DS}=\frac{162X}{\left(4300-42X\right)},$$

where X is the sample acetylation content (%).

### 2.6. Calculation of Cellulose Triacetate Yield

The yield of the product (Y) was computed using Equation (3) with regard to CTA.

(3) $$Y\left(\%\right)=\frac{m_{p}}{\left(\frac{m}{162}\times 288\right)}\times 100\%,$$

where m is the cellulose quality of homemade corn stover (g), m_p_ is the quality of cellulose triacetate (g), 162 is the molar weight of an anhydroglucose unit, and 288 is the molar weight of one CTA unit.

### 2.7. Fourier-Transform Infrared Analysis (FTIR)

To determine the composition of the IL-POM catalyst and the products, the infrared spectra of the IL-POM catalyst, CTA, and CSC were registered using an FTIR spectrophotometer (Perkin Elmer Inc., Waltham, MA, USA) (KBr pellets). Scans were taken over a spectral range of 4000–400 cm^−1^ with 4 cm^−1^ wavenumber resolution and an average of 64 scans.

### 2.8. ^1^H- and ^13^C-NMR Spectroscopy

The ^1^H- and ^13^C-NMR spectra were recorded using a Bruker AVANCE III 600M spectrometer (Bruker, Germany) with D_2_O as the solvent. The data were processed using MestReNova 6.1.1 software.

### 2.9. Scanning Electron Microscopy (SEM)

The morphology of the CTA and corn stover cellulose was examined using SEM (SN-3400, Hitachi, Japan) after sputtering the samples with a gold–palladium mixture under vacuum. All SEM experiments were carried out at an accelerating voltage of 15.0 kV.

### 2.10. Thermal Gravimetric Analysis (TGA)

Thermal gravimetric analysis was carried out using a thermogravimetric analyzer (SDTQ600, TA Instruments, New Castle, DE, USA). The samples were heated from 30 °C to 700 °C with a heating rate of 10 °C/min. The whole process was conducted under nitrogen atmosphere (with a flow rate of 100 mL/min).

### 2.11. Determination of Degree of Polymerization

#### 2.11.1. Determination of Degree of Polymerization of Corn Stover Cellulose

According to Whistler [28], the average degree of polymerization (DP) of CSC was measured using a stopwatch to measure the flow time of the solution and a conventional Ubbelohde viscometer. First, CSC (0.2 g) and distilled water (25 mL) were placed in a 100 mL conical flask and left for 2 h to completely swell. A 0.5 M copper diethylene amine (CED) solution (25 mL) was added and shaken until the CSC was completely dissolved. A conical flask without CSC was used as the blank. Then, the solution intrinsic viscosity was determined using a Ubbelohde viscometer. The CSC degree of polymerization was then calculated on the basis of the intrinsic viscosity values (Equations (4)–(7)).

(4) $$\eta_{r}=\frac{\eta }{\eta_{0}}=\frac{t}{t_{0}},$$

(5) $$\eta_{\mathrm{sp}}=\eta_{r}-1,$$

(6) $$\mathrm{lg}\left[\eta \right]=\mathrm{lg}\left(\frac{\eta_{\mathrm{sp}}}{C}\right)-K_{1}\left[\eta \right]C,$$

(7) $$\left[\eta \right]=K_{2}D_{p}^{\alpha },$$

where η_sp_ is the specific viscosity, η_r_ is the relative viscosity, [η] is the intrinsic viscosity, C is the CSC concentration, η and η_0_ are the viscosity of the CSC solution and solvent at the same temperature, respectively, and t and t_0_ are the outflow time of the CSC solution and solvent in the capillary, respectively; K_1_ = 0.13, K_2_ = 1.7 g/mL, and α = 0.8.

#### 2.11.2. Determination of Degree of Polymerization of Cellulose Acetate

The average degree of polymerization (DP) of CTA was measured according to ASTM Designation D871-96 [27]. First, the CTA sample (0.26 g, dried to constant weight at 105 °C) was placed in a 250 mL flat-bottom flask. The methanol/dichloromethane solvent (1:9, *v*/*v*, 100 mL) was precisely added and stirred at 25 °C ± 0.1 °C for 30 min at a constant speed until the sample was completely dissolved. Then, the solution intrinsic viscosity was determined using a Ubbelohde viscometer. The CTA degree of polymerization (DP) was then calculated on the basis of the intrinsic viscosity values (Equations (4), (8), and (9)).

(8) $$\left[\eta \right]=\left(\frac{3}{C}\right)\left[\mathrm{anti}\log\left(\log\frac{\eta_{r}}{3}\right)-1\right],$$

(9) $$D_{p}=147{\left[\eta \right]}^{1.2},$$

where η_r_ is the relative viscosity, [η] is the intrinsic viscosity, C is the CSC concentration, η and η_0_ are the viscosity of the CSC solution and solvent at the same temperature, respectively, and t and t_0_ are the outflow time of the CSC solution and solvent in the capillary, respectively.

### 2.12. Solubility

First, 0.1 g of dried product was weighed and put into a beaker with an appropriate amount of solvent. The product was stirred at a constant speed for 60 min to observe its dissolution. The solvents included acetic acid, acetone, dichloromethane (DCM), 1,4-dioxane, and dimethyl sulfoxide (DMSO).

### 2.13. Statistical Analysis

All quantitative data were expressed as the mean ± standard deviation. Variance analysis was based on Duncan’s multiple comparison test, and the significance level was set at 0.05.

## 3. Results and Discussion

### 3.1. Structural Characterization of Synthesized IL-POM Catalysts

**Figure 1:**
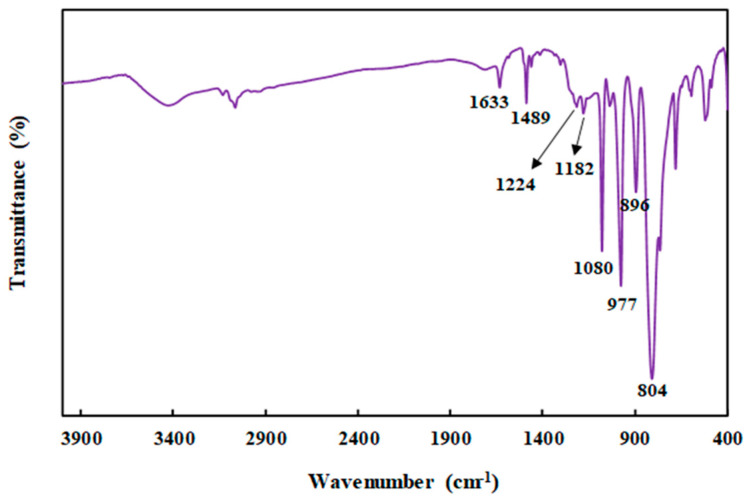
FTIR spectrum of [PyPS]_3_PW_12_O_40_ catalyst.

**Figure 2:**
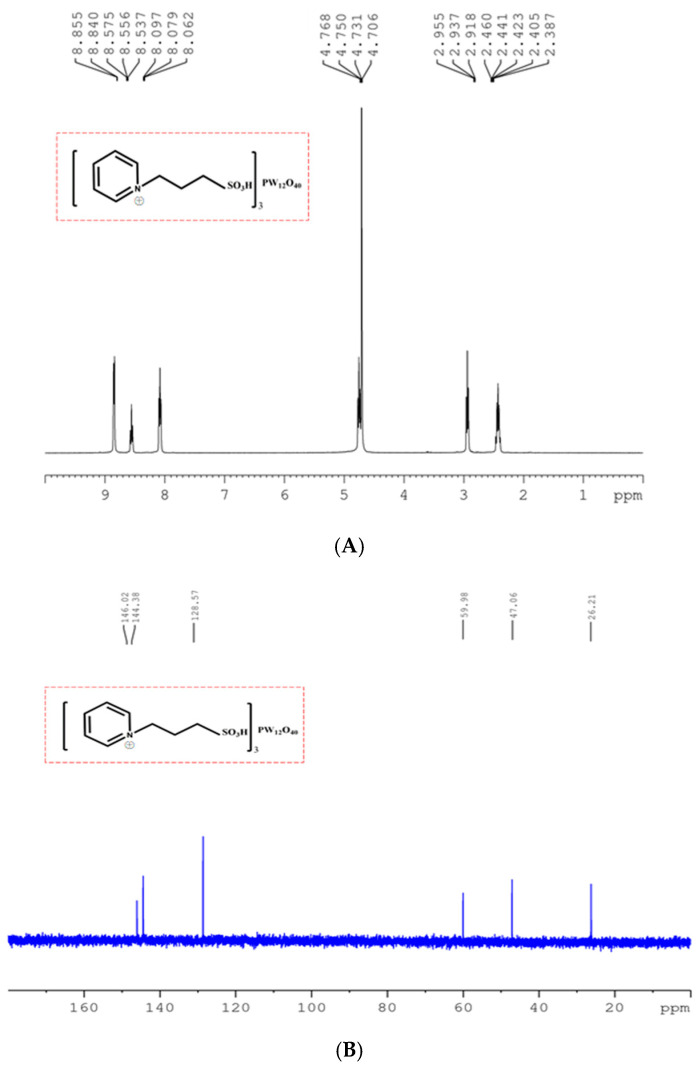
(**A**) ^1^H-NMR of [PyPS]_3_PW_12_O_40_, (**B**) ^13^C-NMR of [PyPS]_3_PW_12_O_40_.

The synthesized IL-POM catalysts were characterized by FTIR, ^1^H-, and ^13^C-NMR spectroscopy. As displayed in Figure 1, the characteristic absorptions of the pyridinium cation (1633, 1488, 1124, and 1181 cm^−1^) and the Keggin heteropolyacid anion (1080, 978, 896, and 804 cm^−1^) can be observed clearly in the spectrum of spent [PyPS]_3_PW_12_O_40_ [19]. The ^1^H-NMR and ^13^C-NMR chemical shifts of [PyPS]_3_PW_12_O_40_ were consistent with those reported in the literature [19,26], indicating that the synthesized catalyst had the organic cations and inorganic heteropolyanion structures of the target ionic liquid. ^13^H-NMR (600 MHz, D_2_O): δ 2.423 (m, 2H), 2.937 (t, 2H), 4.768 (t, 2H), 8.079 (t, 1H), 8.556 (t, 1H), 8.855 (d, 1H) (Figure 2A); ^13^C-NMR (600 MHz, D_2_O): δ 26.21, 47.06, 59.98, 128.57, 144.38, 146.02 (Figure 2B).

### 3.2. Optimization of CTA Synthesis Conditions Catalyzed by IL-POM

Cellulose acetate is prepared by acetylation of cellulose, and the acetylation process is mostly completed in a typical heterogeneous system. It depends on the accessibility of cellulose and the sensitivity of cellulose molecules to acetylation. The catalyst content, acetylation time, and temperature are important aspects of the variable degree of acetylation and acetylated CTA’s physical structure. Hence, the acetylation conditions of CTA were optimized. The DS and yield (%) were calculated for each product, and the results are presented in Table 1.

#### 3.2.1. Optimization of the Reaction Conditions (Catalyst Content)

Table 1 depicts the effect of changing the percentage of catalyst (IL-POM) on the DS and yield (%) of CTA at 140 °C for 45 min. The yield of CTA increased first and then remained stable when the content of IL-POM increased from 0.03 g to 0.08 g. The degree of substitution of CTA remained stable at about 2.95 with the increase in catalyst. Additionally, the yield of CTA was zero when the amount of IL-POM was 0.02 g, which indicated that no substitution reaction occurred under these conditions. Therefore, the minimum content of IL-POM in this work was set at 0.03 g.

It can be seen from Table 1 that the yield of CTA increased by nearly 17%, when the content of IL-POM increased from 0.03 g (62.46%) to 0.04 g (79.27%). However, upon increasing the content of ILP-POM, the CTA yield changed little and remained basically stable. The reason for this phenomenon was that, when the content of IL-POM reached 0.04 g, the central site of the acyl carbon in the acetic anhydride molecule was activated to form a highly reactive acylating agent, which then underwent an acetylation reaction with hydroxyl groups in the cellulose molecule [14,29]. The acetylation reaction presented a critical point when the content of IL-POM exceeded 0.04 g, such that the CTA yield did not change significantly with the increase in catalyst.

As the content of the catalyst increased (0.03~0.08 g), the degree of substitution increased from 2.94 to 2.95, and then stabilized at around 2.95. In this work, the acetylation process was heterogeneous and homogeneous. Firstly, the heterogeneous reaction made the CSC dissolve in the solvent (acetic acid). The presence of acetic acid changed the microcrystalline structure of cellulose, improved the accessibility of acetic anhydride and cellulose, and played an important role in the activation of cellulose acetylation. Secondly, the CSC underwent a homogeneous reaction in the solvent. The Brønsted acidic IL-POM activated the carbonyl group of the acetic anhydride, protonated the carbonyl group of the acetic anhydride, and made the latter more reactive with the hydroxyl groups of cellulose. During the reaction, the new cellulose surface was continuously exposed to acetylation [30]. Meanwhile, the crystal structure of cellulose was rapidly destroyed at 140 °C, which shortened the heterogeneous reaction time and promoted the acetylation reaction, thus improving the degree of substitution of products. The CTA prepared in this study, with a high degree of substitution, has excellent application prospects. Generally speaking, considering the factors such as CTA yield, degree of substitution, and cost, the optimal content of IL-POM in this reaction was 0.04 g.

#### 3.2.2. Optimization of the Reaction Conditions (Temperature)

The reaction temperature had an important effect on CTA yield. As shown in Table 1, the yield (%) increased and DS remained stable as the temperature increased. In particular, when the temperature was 120 °C, CTA could not be detected when other reaction conditions were unchanged, indicating that the reaction would not proceed. The CTA yield increased from 0% to 44.01% with the temperature increasing from 120 °C to 130 °C. The yield of CTA was as high as 79.27%, with the temperature rising to 140 °C. Then, as the temperature continued to rise, the CTA yield changed little. There are two reasons for the above phenomenon. On one hand, 120 °C was not high enough for IL-POM catalytic activity in the acetylation reaction; hence, the reaction was ineffective. When the temperature rose to 130 °C, the central site of the acyl carbon in the acetic anhydride molecule was activated to form an efficient acylating agent, which cooperated with the efficient catalysis of IL-POM to produce an acetylation reaction with the hydroxyl group of the cellulose molecule. On the other hand, with a further increase in temperature, the destruction rate of cellulose crystalline region was accelerated, the movement frequency between molecules was increased, the reaction efficiency was improved, and the CTA yield was improved. However, after the temperature reached 140 °C, the whole acetylation reaction was basically saturated, and the CTA yield remained stable.

The degree of substitution of CTA fluctuated slightly between 2.94 and 2.95, with the increase in reaction temperature. Due to the short heterogeneous time of the whole reaction, as well as the relatively long homogeneous reaction time and the high catalytic activity of IL-POM, the substitution degree of CTA was ideal. Considering CTA yield and reaction energy consumption, the best reaction temperature was 140 °C.

#### 3.2.3. Optimization of the Reaction Conditions (Time)

Table 1 shows the effect of reaction time on CTA yield and DS. With the increase in reaction time, the yield of CTA first increased and then remained unchanged. However, the DS of CTA remained between 2.94 and 2.95, without significant change. With the increase in reaction time from 15 to 45 min, the yield of CTA increased by nearly 30%. This was because the acetylation reaction between cellulose molecules on the surface layer and acetic anhydride occurred first after the reaction started. With the extension of time, adsorption and diffusion effects between cellulose molecules and acetic anhydride were produced, thereby improving the reaction efficiency and increasing the yield of CTA. When the reaction time increased to 60 min, the yield of CTA was not improved significantly. This could be due to the reduction in reaction sites on the surface of the cellulose molecules. Accordingly, 45 min was selected as the most appropriate condition with high CTA yield and ideal DS.

### 3.3. Properties of Synthesized CTA

#### 3.3.1. FTIR Analysis

**Figure 3:**
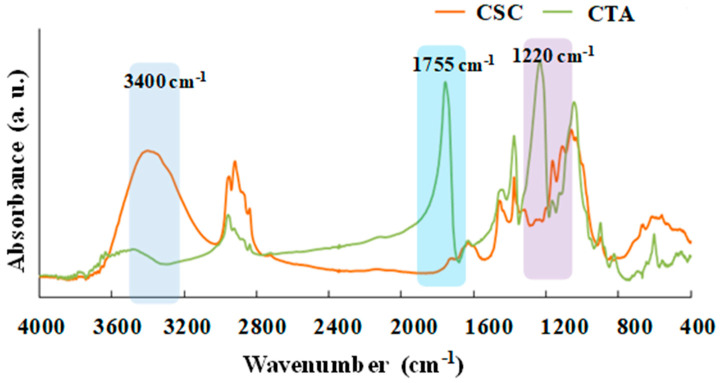
Infrared absorbance spectra of CSC and CTA.

The acetylated products were characterized by FTIR spectroscopy to observe the ester peaks and hydroxyl group absorptions. Figure 3 illustrates the FTIR spectra of corn stover cellulose (CSC) and the synthesized CTA. Three major changes were observed in the spectrum of CTA as compared to CSC. Firstly, a significant decrease in the hydroxyl (–OH) stretching band at 3400 cm^−1^ in the spectrum of CTA was found, indicating that –OH on the cellulose molecule was almost completely replaced. The remaining insignificant peak at 3400 cm^−1^ could be attributed to the a small amount of water in the sample. There was an increase in the carbonyl (C=O) stretching band at 1755 cm^−1^, which is a characteristic peak of acetyl groups. Lastly, an increase in carbon–oxygen (C–O) stretching vibration at 1220 cm^−1^ represented a characteristic peak of ester groups [13]. The above results illustrated that an acetylation reaction occurred, acetyl groups were formed in the product, and CTA was successfully synthesized. The disappearance of peaks in region 1840–1760 cm^−1^ and at 1700 cm^−1^ in the spectrum (Figure 3) indicated that the product was free of the unreacted acetic anhydride and the byproduct acetic acid [31].

#### 3.3.2. SEM Analysis

**Figure 4:**
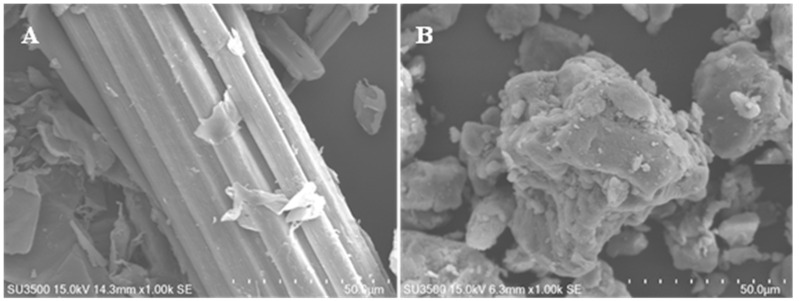
SEM images of CSC (**A**) and CTA (**B**).

The microscopic morphology of corn stover cellulose and CTA was characterized by SEM (Figure 4). As shown in Figure 4A, the surface of corn stover cellulose was smooth and had a regular bundle-like fiber structure. The fibers appear to be ordered by several microfibers of cellulose aligned in the direction of the major axis of corn stover, as suggested in [25,32]. However, the product CTA obtained after the acetylation reaction presented a cluster-like structure of different sizes, and the surface became rough and loose (Figure 4B). The acetylation reaction of cellulose was a homogeneous process in the presence of IL-POM [33]. Cellulose macromolecules gradually dissolved in acetic acid, and it was clear that the original structure of cellulose was completely destroyed. At the end of the acetylation reaction, the products were precipitated in deionized water and agglomerated into granular structures of varying sizes.

#### 3.3.3. TG Analysis

**Figure 5:**
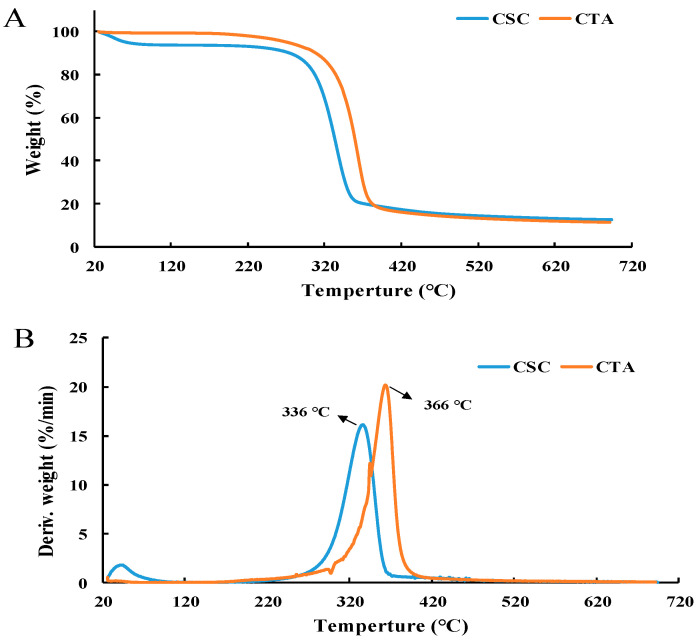
TGA (**A**) and DTG (**B**) curves of CSC and CTA.

The effect of acetylation on the thermal properties of CSC was examined by thermogravimetric analysis (TG) in the temperature range from 25 °C to 700 °C. The TG and DTG curves and correlation results obtained for CSC and CTA are shown in Figure 5 and Table 2. 

In general, the degradation reactions observed in CSC and CTA curves (Figure 5) occurred at a specific thermal degradation stage and in a narrow temperature range, indicating that all samples were composed mainly of a unique component. As for CSC, two regions of weight loss were observed. The first region of 40.43 °C to 63.66 °C was attributed to water evaporation. The major weight loss (79.69%) happened in the second region ranging from about 307.61 °C to 354.09 °C, related to the decomposition of cellulose [25]. The major weight loss (86.92%) of CTA happened in the region ranging from about 339.26 °C to 379.73 °C, owing to the decomposition of the breakdown of the acetyl group in cellulose triacetate [32]. The thermal degradation temperatures were considered to start at 307.61 °C for CSC and 339.26 °C for CTA; the highest rate of weight loss of CSC occurred at 336.00 °C, while that of CTA happened at 366.00 °C, as shown in Table 2 and Figure 5B. This clearly indicated that the thermal stability of the CTA was significantly higher than that of CSC, probably due to its higher DS. This can be explained by the fact that, as DS increased, more acetyl groups were introduced onto the C2 and C3 carbons of the anhydroglucose unit, which required more energy when compared to the less sterically hindered C_6_COOCH_3_ group, thus increasing the apparent activation energy for thermal decomposition, as well as the thermal stability [14,34]. As reported by Alves et al. [32], the maximum degradation of cellulose acetate corresponding to 87% mass loss (at 385 °C) was higher than that of sorghum straw (67% at 339 °C), pulp (75% at 371 °C), and bleached pulp (80% at 369 °C). Furthermore, as evidenced by Cao et al. [33], the thermal stability of CA samples increased as the DS value increased. Furthermore, as can be seen from Table 2, the mass residual rates of CSC and CTA after 700 °C were 12.58% and 11.37%, respectively, corresponding to the residual rate of carbon.

#### 3.3.4. Degree of Polymerization of CTA

The degree of polymerization of corn straw cellulose prepared in this work was 276.06. The degree of polymerization of CTA prepared under the optimum reaction conditions (cellulose (0.4 g), acetic acid (9.0 mL), acetic anhydride (2.0 mL), IL-POM (0.04 g), 140 °C, 45 min) was 91.74, which is lower than that of cellulose acetate (204) sold on the market. The main reason was that the pretreatment temperature of corn straw was 130 °C, and the catalytic degradation of peracetic acid led to the destruction of cellulose macromolecules to a certain extent. At the same time, the temperature of 140 °C would also deconstruct the molecular structure of cellulose during acetylation. Therefore, CTA with a reduced polymerization degree was obtained.

#### 3.3.5. Solubility of CTA

The solubility of the synthesized CTA in common organic solvents (acetic acid, acetone, dichloromethane (DCM), 1,4-dioxane, and dimethyl sulfoxide (DMSO)) is presented in Table 3. As shown, the synthesized CTA was soluble in acetic acid, DCM, 1,4-dioxane, and DMSO at room temperature. However, the synthesized CTA was insoluble in acetone. Since acetyl groups are more hydrophobic than hydroxyl groups, the replacement of the hydroxyl groups in cellulose with acetyl groups enhanced its solubility in organic solvents [35]. The solubility of the synthesized CTA in organic solvents was consistent with that of CTA with a typically high degree of substitution [12]. This indicated that the synthesized product prepared in this work was CTA with a high degree of substitution. These observations are partly consistent with the literature [36]. Furthermore, the research results provide a certain reference value for the selection of solvents in the follow-up production and practical application of corn stover-based CTA. 

## 4. Conclusions

The proposed work mainly focused on exploring a green pathway for utilizing high-potential biomass materials. This method uses low concentrations of peracetic acid and maleic acid (PAM) to enrich cellulose from corn stover in one step. The CSC was successfully catalyzed to synthesize CTA using IL-POM. The yield of CTA was greatly affected by the amount of IL-POM, reaction temperature, and reaction time, while the degree of substitution of CTA was less affected. The yield of CTA could reach 79.27%, and the degree of substitution was 2.95. The morphology, structure, and thermal stability of CTA were verified using SEM, FTIR, and TGA, respectively. The micromorphology of CTA presented a cluster-like structure with different sizes in the SEM studies. FTIR results showed that the cellulose hydroxyl group was substituted by an acetyl group, and there was no residual acetic anhydride. The thermal analysis revealed that CTA was more thermally stable than CSC, which was attributed to the increase in acetyl content. Furthermore, CTA had excellent solubility in organic solvents such as acetic acid, DCM, 1,4-dioxane, and DMSO, providing a reference value for the practical application of CTA.

The findings reveal a new pathway for the green and efficient synthesis of CTA, exploring the utilization of agricultural residue and green chemistry. The successful preparation of CTA can effectively promote the maturity of corn stover biorefinery, with application for the large-scale development of biobased industries with low carbon emissions.

## Figures and Tables

**Table 1:** Effects of reaction temperature, IL-POM content, and reaction time on the yield and degree of substitution of CTA.

Factors		Yield (%)	Degree of Substitution
Temperature (°C)	130	44.01 ± 2.65 ^b^	2.94 ± 0.01 ^a^
	140	79.27 ± 0.02 ^a^	2.94 ± 0.00 ^a^
	150	80.34 ± 0.26 ^a^	2.95 ± 0.01 ^a^
IL-POM contents (g)	0.03	62.46 ± 2.28 ^b^	2.94 ± 0.01 ^a^
	0.04	79.27 ± 0.02 ^a^	2.94 ± 0.00 ^a^
	0.06	81.08 ± 0.16 ^a^	2.95 ± 0.01 ^a^
	0.08	81.09 ± 0.12 ^a^	2.94 ± 0.00 ^a^
Time (min)	15	49.62 ± 2.86 ^c^	2.94 ± 0.01 ^a^
	30	68.69 ± 0.10 ^b^	2.94 ± 0.00 ^a^
	45	79.27 ± 0.02 ^a^	2.94 ± 0.01 ^a^
	60	79.77 ± 1.26 ^a^	2.95 ± 0.00 ^a^

Values are given as the mean ± SD of triplicate determinations; a~c indicates that different letters in the same factor differ significantly (*p* < 0.05).

**Table 2:** Thermogravimetric results for corn stover cellulose (CSC) and cellulose triacetate (CTA).

Sample	T_onset_ (°C)	T_max_ (°C)	T_end_ (°C)	W_loss_ (%)	W_residual_ (%)
CSC	307.61	336.00	354.09	79.69	12.58
CTA	339.26	366.00	379.73	86.92	11.37

T_onset_: onset temperature, T_max_: temperature of maximum weight loss rate, T_end_: end temperature.

**Table 3:** The solubility of CTA in common solvents.

Solvents	Solubility ^x^
Acetic acid	+
Acetone	−
Dichloromethane	+
1,4-Dioxane	+
Dimethyl sulfoxide	+

^x^ Solubility of product in common solvents: “+” represents soluble, and “−” represents insoluble.

## Data Availability

Not applicable.
